# Associations of Red Cell Distribution Width With Coronary Artery Calcium in the General Population

**DOI:** 10.1177/00033197211052124

**Published:** 2021-11-24

**Authors:** Jingxue Pan, Yan Borné, Isabel Gonçalves, Margaretha Persson, Gunnar Engström

**Affiliations:** 1 Department of Clinical Sciences in Malmö, 5193 Lund University, Malmö, Sweden; 2 Department of Cardiology, Skåne University Hospital, Sweden

**Keywords:** red cell distribution width, coronary calcification, atherosclerosis, general population

## Abstract

Red cell distribution width (RDW) is a measure of the variability of erythrocyte volumes. RDW has been associated with incidence of cardiovascular diseases. However, the underlying mechanisms for the increased cardiovascular risk are still unclear. This study aimed to examine associations of RDW and coronary atherosclerosis in the general population. Computed tomography was performed and RDW was measured in fresh blood from 5772 subjects (aged 50–64 years) from the Swedish CArdioPulmonary bioImage Study (SCAPIS). Multinomial logistic regression was conducted to examine the associations between RDW and coronary artery calcium score (CACS). A total of 3902 (67.6%) individuals had low CACS (≤10), 18.6% had moderate CACS (>10 and ≤100) and 13.8% had high CACS (>100). The proportion with high CACS was 11.7%, 12.7%, 13.7% and 18.3%, respectively, in quartile 1–4 of RDW. After controlling for traditional risk factors, there were significant associations between RDW and high CACS: odds ratio = 1.51, 95% confidence interval: 1.18–1.94, and *P* = .001, for 4^th^ vs 1^st^ quartile of RDW. Elevated RDW is associated with coronary artery calcification in the middle-aged general population. RDW could be a reproducible and easily assessable biomarker of coronary calcification and cardiovascular risk.

## Introduction

Red cell distribution width (RDW) reflects the heterogeneity of the volumes of circulating erythrocytes and is often used to diagnose different subtypes of anaemia.^1^ Recently, RDW has drawn increasing attention as a potential risk factor for incidence of cardiovascular (CV) diseases,^2,3^ including myocardial infarction (MI),^4^ fatal coronary events^5^ and heart failure (HF).^6^ High RDW was also associated with increased mortality among patients with coronary artery disease (CAD)^7^ and HF.^8^ Little is known about the underlying mechanisms for the increased CV risk. However, it is noteworthy that a big population-based study found that high RDW substantially increased the risk of coronary events with fatal outcomes, while no significant relationship was found for non-fatal MI.^5^

Atherosclerosis is the principal cause of CV events. Although growing evidence supports that high RDW is associated with an unfavourable CV risk profile and higher CV disease mortality in various populations, the relation between RDW and coronary atherosclerosis has not been sufficiently investigated. Coronary artery calcium score (CACS)^9^ is a widely known and specific marker of coronary atherosclerosis. It offers better discrimination for atherosclerotic CV events than a broad range of conventional CV risk factors^10^ and reflects the coronary atherosclerosis burden.^11^ CACS has also been shown to improve risk stratification^12^ for individuals at intermediate risk of CV diseases. Associations between RDW and CACS have been reported in patients with suspected or known CAD who underwent computed tomography (CT).^13,14^ It is unknown, however, whether RDW is associated with coronary calcification in the general population.

The aim of study was to investigate this gap in knowledge by evaluating the associations between RDW and CACS in the population-based Swedish CArdioPulmonary bioImage Study (SCAPIS) after controlling for other risk factors.

## Methods

### Study Population

The SCAPIS study is a collaboration between six Swedish universities and university hospitals (Gothenburg, Malmö, Stockholm, Uppsala, Linköping and Umeå) with the purpose of studying cardio-pulmonary diseases in a large population-based cohort from six areas in Sweden. A total of 30,154 men and women (50–64 years of age) participated.^15^ Analysis of RDW was included in the baseline examinations for participants examined in Malmö. A total of 6251 individuals were examined at this site (participation rate 53%).

**Figure 1.**
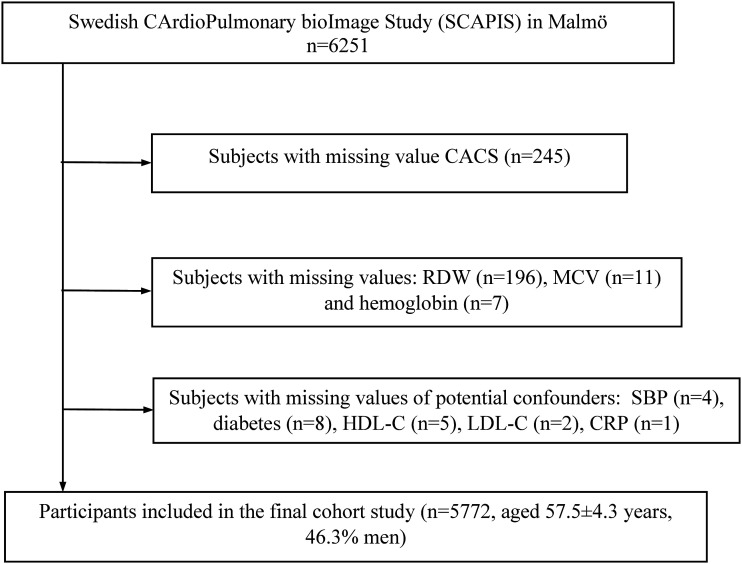
Study flow chart. SCAPIS, Swedish CArdioPulmonary bioImage Study; CACS, coronary artery calcium score; RDW, red cell distribution width; MCV, mean corpuscular volume; SBP, systolic blood pressure; HDL-C, high-density lipoprotein cholesterol; LDL-C, low-density lipoprotein cholesterol; CRP, C-reactive protein.

The subjects completed a self-administered questionnaire and underwent a health examination, which was conducted during 2014 to 2018. After exclusion of missing values of CACS (n = 245), RDW (n = 196) and CV risk factors (n = 38), a total of 5772 participants were included in the present study (Figure 1).

The study complied with the Declaration of Helsinki. Written informed consent was obtained from all participants. SCAPIS has been approved as a multicentre trial by the ethics committee at Umeå University (February 21, 2011). The present analysis has been approved by Lund University ethical review board (2016-1031; 2018-551).

### Coronary Artery Calcification Assessment

CT was performed using equipment from Siemens (Definition Flash 2x128 slice, stellar detector, 4D-Care dose, Care-kV and SAFIRE, Forchheim, Germany).^15^ CAC images were obtained using electrocardiogram-gated non-contrast CT imaging at 120 kV. A β-blocker (metoprolol 50 mg orally) was given for control of heart rate. All non-contrast image sets were reconstructed and CAC were identified and scored using the syngo.via calcium scoring software (Siemens Healthineers, Solna, Sweden). CACS was calculated as the sum of calcium content in each coronary artery. The area of calcification of each 3 mm slice was multiplied with an intensity factor with the scoring system according to Agatston.^16^ The subjects were categorized in three groups of CACS, according to previously proposed categories of risk^17,18^: low CACS (≤10), moderate CACS (>10 and ≤100) and high CACS (>100).

### Examinations

Smoking was categorized as current smokers and non-smokers (i.e. former or never-smokers) from questionnaires. Information about anti-hypertensive medication, anti-diabetic medication, lipid lowering medication and erythropoietin analogues were obtained from questionnaires. Supplements intake information regarding vitamin B12, folic acid and iron was obtained from questionnaire. Subjects with rheumatic diseases (such as rheumatic arthritis, ankylosing spondylitis, psoriasis arthritis, systemic lupus erythematosus or Sjögren’s disease) were ascertained from self-reported questionnaire.

A fasting venous blood sample was collected for analysis of lipids, creatinine, glucose, haemoglobin (HB) and C-reactive protein (CRP). RDW was measured using a Sysmex XN-10 counter (www.sysmex.com). RDW was calculated as the width (fL) of the erythrocyte distribution curve at the relative height of 20% above the baseline.^1^ Anaemia was defined as HB <120 g/L in women and <130 g/L in men.

Body weight was measured on a digital scale with subjects dressed in light indoor clothing without shoes. Body height was measured and approximated to the nearest cm. Body mass index (BMI) was calculated as body weight/height^2^ (kg/m^2^). Systolic blood pressure (SBP) was measured in supine position twice in each arm with an automatic device (Omron M10‐IT. Omron Health care Co. Kyoto. Japan). Mean SBP from the arm with the higher mean SBP was used in the analysis. The participants’ kidney function was calculated by the estimated glomerular filtration rate (eGFR, ml/min/1.73 m^2^).^19^

### Test-Retest Reliability of RDW

A total of 154 men and women were examined with repeated blood tests after 1 year (±1 month). RDW was available for 150 of them. This subgroup was used to study variability of RDW over time.

### Statistical Analyses

Continuous variables were expressed as means with standard deviations (means ± SDs) or median (interquartile range, IQR) and categorical data were presented as numbers and percentages (n, %). The values of RDW were divided into quartiles and the first quartile (Q1) reflects the erythrocytes that vary the least in size and the fourth quartile (Q4) varies the most in size. CRP and glucose levels were base-e logarithmically transformed for statistical analyses, due to skewed distribution. CRP values below lower limit of quantification (LLQ, i.e. .6 mg/L) were replaced by LLQ/1.414 mg/L. Continuous variables were compared using the analysis of variance (ANOVA) and the chi-square test was used for categorical variables.

The association between RDW (independent variable) and CACS (dependent variable) was examined by multinomial logistic regression analysis with multiple adjustments. Odds ratio (OR) with 95% confidence intervals (CIs) were reported. Model 1 was adjusted for age and sex and model 2 was adjusted for CV risk factors (i.e. age, sex, SBP, BMI, ln-transformed CRP, current smoking, ln-transformed glucose, high-density and low-density lipoprotein cholesterol (HDL-C and LDL-C), anaemia, anti-hypertensive medication, lipid lowering medication, anti-diabetic medication and eGFR). RDW was also tested as a continuous variable (per 1 SD, i.e. 3.08 fL).

Interactions between RDW (per 1 SD increment) and other CV risk factors were tested using multiplicative interaction terms in the logistic regression model. Since RDW correlated significantly with mean corpuscular volume (MCV) (tested by Spearman’s correlation coefficient, r), we examined potential effect modification by MCV, using a multiplicative interaction term between RDW and MCV above or below median and in a stratified analysis of high and low MCV. We also assessed the associations between RDW and CACS stratified by sex, smoking and median values of age, SBP and HDL-C, respectively.

Sensitivity analyses of the associations between RDW and CACS were conducted after excluding individuals with anaemia, after excluding individuals with rheumatic diseases and after exclusion of participants with supplements intake of vitamin B12, folic acid, iron and treatment with erythropoietin analogues, respectively.

We performed receiver operating characteristic (ROC) curve analysis to estimate the model discrimination for high CACS in a model including all risk factors in model 2, with and without RDW.

Intra-class correlation coefficients (ICCs) were calculated to assess test–retest variability of RDW after 1 year. A two-way mixed model design was used to calculate absolute agreement between measurements.

A 2-tailed *P* < .05 was considered significant. All the analyses were performed using IBM SPSS Statistics V.27 (www.spss.com).

## Results

### Population Characteristics

**Figure 2.**
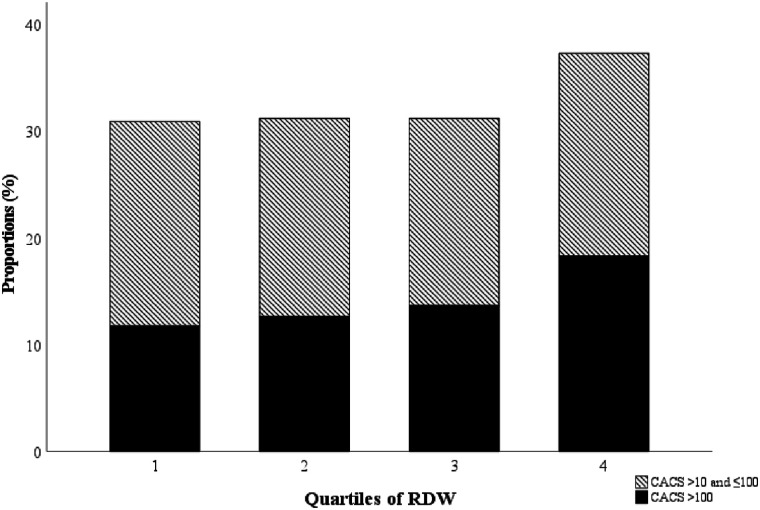
Proportions of moderate and high CACS across quartiles of RDW. CACS, coronary artery calcium score; RDW, red cell distribution width.

A total of 5772 individuals were included in this study. The CV disease risk factors are presented across the quartiles of RDW in Table 1. Among the whole population, 67.6% had low CACS (≤10), 18.6% had moderate CACS (>10 and ≤100) and 13.8% had high CACS (>100). The proportion of participants with high CACS (>100) was 11.7%, 12.7%, 13.7% and 18.3%, respectively, in quartile 1–4 of RDW (Figure 2). RDW (mean ± SD) was 42.0 ± 3.1 fL in men and 42.6 ± 3.1 fL in women, respectively. High RDW was associated with age, female sex, current smoking, SBP, CRP, MCV, HDL-C and with low BMI and haemoglobin (Table 1).

**Table 1.** Characteristics of the study population across quartiles of RDW.

	Q1 (lowest) (n = 1661)	Q2 (n = 1595)	Q3 (n = 1332)	Q4 (highest) (n = 1184)	*P*
RDW (fL)	39.0 ± 1.2	41.5 ± 0.5	43.4 ± 0.5	46.8 ± 2.3	<.001
Anaemia (n, %)	31(1.9)	28(1.8)	33(2.5)	69(5.8)	<.001
Haemoglobin (g/L)	144.7 ± 12.1	143.2 ± 11.5	142.0 ± 11.9	139.5 ± 12.7	<.001
MCV (fL)	86.3 ± 3.7	88.9 ± 3.0	90.3 ± 3.5	92.4 ± 4.6	<.001
Age (years)	56.6 ± 4.2	57.4 ± 4.3	57.7 ± 4.2	58.4 ± 4.4	<.001
Men (n, %)	868(52.3)	760(47.6)	582(43.7)	464(39.2)	<.001
BMI (kg/m^2^)	27.5 ± 4.2	27.2 ± 4.4	27.1 ± 4.7	27.1 ± 5.2	.032
Systolic blood pressure (mmHg)	123.4 ± 16.2	122.8 ± 16.7	123.0 ± 16.7	125.1 ± 17.5	.001
Current smoking (n, %)	170(10.2)	249(15.6)	255(19.1)	323(27.3)	<.001
CRP (mg/L)	1.1(.4; 2.3)	1.1(.4; 2.4)	1.2(.4; 2.7)	1.3(.7; 3.0)	<.001
eGFR^a^ (ml/min/1.73 m^2^)	85.6 ± 11.8	84.3 ± 12.4	84.4 ± 12.2	85.5 ± 13.2	.003
HDL-C (mmol/L)	1.5 ± 0.5	1.7 ± 0.5	1.7 ± 0.5	1.8 ± 0.6	<.001
LDL-C (mmol/L)	3.6 ± 0.9	3.6 ± 1.0	3.6 ± 0.9	3.6 ± 1.0	.498
Glucose (mmol/L)	5.4(5.0; 5.8)	5.3(4.9; 5.8)	5.3(4.9; 5.8)	5.3(4.9; 5.8)	<.001
Rheumatic diseases (n, %)	46 (2.8)	42 (2.6)	53(4.0)	79 (6.7)	<.001
Medication					
Lipid lowering medication (n, %)	127(7.6)	133(8.3)	81(6.1)	109(9.2)	.085
Anti-hypertensive medication (n, %)	314(18.9)	330(20.7)	265(19.9)	263(22.2)	.098
Anti-diabetic medication (n, %)	71(4.3)	71(4.5)	50(3.8)	41(3.5)	.526
Supplements intake for vitamin B12, folic acid and iron (n, %)	36 (2.2)	37 (2.3)	33 (2.5)	77 (6.5)	<.001
CACS (n, %)					<.001
Low CACS (≤10)	1147(69.1)	1097(68.8)	916(68.8)	742(62.7)	
Moderate CACS (>10 and ≤100)	319(19.2)	296(18.6)	234(17.6)	225(19.0)	
High CACS (>100)	195(11.7)	202(12.7)	182(13.7)	217(18.3)	

Values are expressed as mean ± standard deviation (SD) or as median (25^th^ and 75^th^ percentiles) (for skewed distributions) for continuous variables, or n (%) for categorical variables.

Analysis of variance (ANOVA) or Pearson chi-square was used to calculate *P*-value for the association across RDW quartiles.

Abbreviations: RDW, red cell distribution width; Q, quartile; CACS, coronary artery calcium score; MCV, mean corpuscular volume; CRP, C-reactive protein; BMI, body mass index; eGFR, estimated glomerular filtration rate; HDL-C, high-density lipoprotein cholesterol; LDL-C, low-density lipoprotein cholesterol.

^a^Information on eGFR was available for 5770 individuals.

### The Associations Between RDW and CACS

After adjustment for age and sex, CACS (>100) increased significantly with RDW: OR = 1.63, 95% CI: 1.29–2.05, *P* <.001, for 4^th^ vs 1^st^ quartile of RDW. After adjustments for other CV risk factors (model 2), there was still a significant association between RDW and high CACS: OR = 1.51, 95% CI: 1.18–1.94, *P* = .001 (4^th^ vs 1^st^ quartile of RDW) (Table 2). The results were similar when RDW was modelled as a continuous variable (per 1 SD) (OR for CACS>100: 1.19, 95% CI: 1.09–1.29, *P* <.001).

**Table 2.** Associations between RDW and coronary calcium scores.

		Quartiles of RDW				
	CACS	Q1 (n = 1661)	Q2 (n = 1595)	Q3 (n = 1332)	Q4 (n = 1184)	Per 1 SD of RDW
Model 1	Moderate	1	.95(.79−1.14)	.91(.74−1.10)	1.09(.89−1.33)	1.03(.96−1.11)
	High	1	1.02(.81−1.27)	1.12(.89−1.41)	1.63(1.29−2.05)***	1.22(1.13−1.32)***
Model 2	Moderate	1	.96(.80−1.16)	.91(.74−1.11)	1.05(.85−1.30)	1.01(.94−1.09)
	High	1	1.03(.81−1.30)	1.12(.88−1.43)	1.51(1.18−1.94)**	1.19(1.09−1.29)***

Values are odds ratios (95% confidence intervals) obtained from multinomial logistic regression models. RDW in Q1 is the reference category.

Abbreviations: RDW, red cell distribution width; CACS, coronary artery calcium score; Q, quartile; SD, standard deviation.

Model 1: adjustment for age and sex; model 2: adjustment for model 1 + current smoking, body mass index, ln-transformed C-reactive protein, anti-diabetic medication, ln-transformed glucose, high-density lipoprotein cholesterol, low-density lipoprotein cholesterol, systolic blood pressure, anaemia, anti-hypertension medication, lipid lowering medication and estimated glomerular filtration rate.

**: *P* <.01; ***: *P* <.001.

The area under the curve (AUC) statistics for high CACS was .773 (95%CI: .756-.790) for a model including all variables in model 2, except RDW. AUC increased to .776 (95%CI: .760-.793) when RDW was added to the model.

### Effect Modification by Other Risk Factors

**Figure 3.**
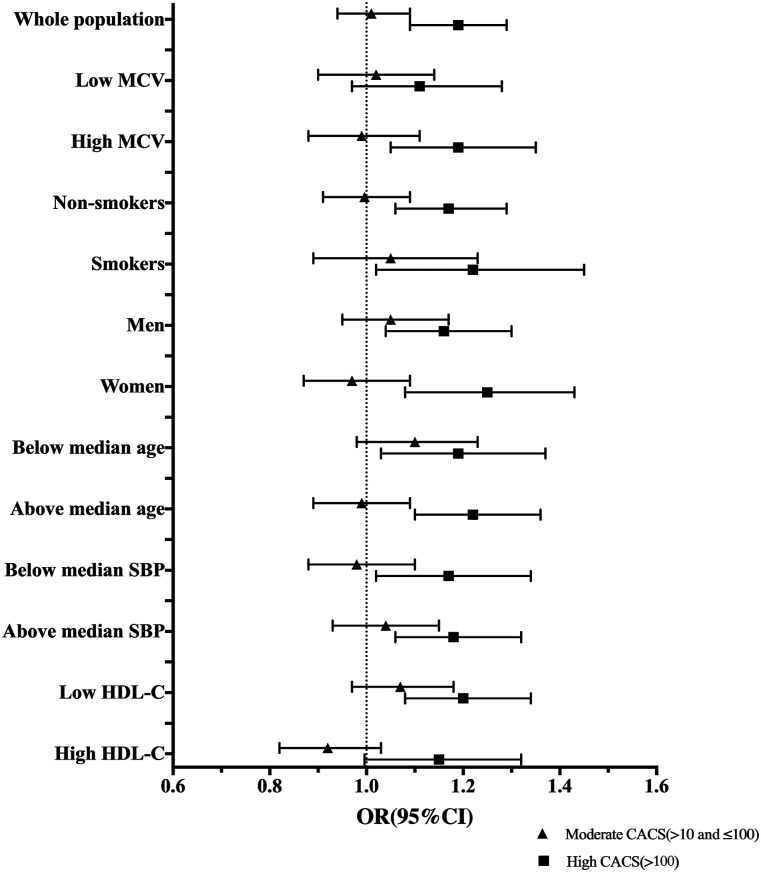
Associations between per 1 SD increase of RDW and CACS in the whole population and different subgroups of cardiovascular risk factors. OR (95% CI) were obtained from multinomial logistic regression models. CACS ≤10 is the reference category. Model was adjusted for age, sex, body mass index, ln-transformed C-reactive protein, high-density lipoprotein cholesterol, low-density lipoprotein cholesterol, systolic blood pressure, anti-hypertension medication, lipid lowering medication, estimated glomerular filtration rate, ln-transformed glucose, current smoking, anaemia and anti-diabetic medication (when appropriate). Median MCV = 89 fL; median age = 57.40 years; median SBP = 122 mmHg; median HDL-C = 1.60 mmol/L. CACS, coronary artery calcium score; MCV, mean corpuscular volume; SBP, systolic blood pressure; HDL-C, high-density lipoprotein cholesterol; OR, odds ratio; CI, confidence interval; SD, standard deviation; RDW, red cell distribution width.

RDW correlated with MCV (r = .57, *P* <.01). Since high RDW could be related to microcytosis as well as macrocytosis, we also explored the associations of RDW and CACS stratified by median of MCV (i.e. 89 fL). We found significant relationships between RDW and high CACS among subjects with high MCV (OR per 1 SD: 1.19, 95% CI: 1.05–1.35, *P* = .007) (Figure 3). For individuals with low MCV, RDW was associated with high CACS in the crude model but not significantly after adjustments for risk factors. There was a significant interaction between RDW and MCV when both were modelled as continuous variables. However, the interaction was not significant when MCV was dichotomized into high and low levels.

To investigate whether smoking explained the association between RDW and CACS, we analysed the associations between RDW and CACS among smokers and non-smokers separately. The associations between RDW and high CACS were significant both in smokers (OR per 1 SD: 1.22, (95% CI: 1.02–1.45), *P* = .028) and non-smokers (OR per 1 SD: 1.17 (95% CI: 1.06–1.29), *P* = .002) (Figure 3). There were no significant interactions between RDW and smoking.

The associations between per 1 SD increase of RDW and CACS in the whole population and different subgroups of CV risk factors are presented in Figure 3.

### Sensitivity Analyses

We excluded individuals with anaemia (n = 161) in a sensitivity analysis. The results were essentially unchanged: OR (95% CI) for high CACS among non-anaemic subjects was 1.53 (1.19–1.97, *P* = .001) for 4^th^ vs 1^st^ quartile of RDW. We also performed a sensitivity analysis excluding individuals who reported supplements intake of vitamin B12, folic acid or iron and one individual treated with an erythropoietin analogue (n = 184). The associations between RDW and high CACS among participants excluding supplements intake were still significant: OR for 4^th^ vs 1^st^ quartile of RDW = 1.476, 95% CI: 1.142–1.909, *P* = .003.

Finally, a sensitivity analysis was performed after exclusion of participants who reported a diagnosis of any rheumatic disease (n = 220). The multivariate-adjusted associations between RDW and high CACS remained significant: OR for 4^th^ vs 1^st^ quartile of RDW = 1.476, 95% CI: 1.143–1.908, *P* = .003.

### Test–Retest Reliability of RDW

The test–retest reliability for RDW was assessed using repeated blood tests after 1 year in a subgroup of 150 men and women. Mean RDW in this group was 41.31 ± 2.93 fL at baseline and 41.89 ± 2.98 fL after 1 year. ICC was .76, 95% CI: .67–.83.

## Discussion

It has previously been reported that high RDW is associated with increased incidence of CV diseases in the general population and that RDW is associated with poor prognosis in patients with established CV disease.^7,8,20,21^ The present study demonstrated that RDW is significantly associated with coronary atherosclerosis, as measured by CACS. This association remained significant after adjustment for CV risk factors. Since smoking is a major determinant of high RDW,^4,5^ it is noteworthy that the relationships were significant both in smokers and non-smokers.

Only a few studies have previously investigated the associations between RDW and coronary artery atherosclerosis.^13,14,22^ Den Harder et al.^13^ found that RDW was associated with higher CACS in patients with suspected or known CAD. Also, Gürel et al.^14^ reported that high RDW was associated with coronary calcification in a sample of consecutive outpatients. Wang et al. demonstrated that RDW correlated with severity of coronary stenosis in CAD patients.^22^ In contrast, Chaikriangkrai et al.^23^ did not find significant associations with CACS in a study triaging chest pain patients without known CAD. RDW was associated with atherosclerosis in the carotid arteries in the Tromsø study.^24^ To our knowledge, there are no big population-based studies of RDW and coronary atherosclerosis.

Atherosclerotic calcification is likely an organized, regulated process, which is related to vascular inflammation^25^ and possibly also with bone formation.^26,27^ It was recently shown that lysed erythrocyte membranes, but not intact erythrocytes, could potentiate osteoblastic differentiation and that lysed erythrocyte membranes could promote vascular calcification in genetically modified mice and human vascular specimens in a nitric oxide-dependent way.^28^ This could be a possible link between increased turnover of the red cells and atherosclerosis. It has also been suggested that intraplaque haemorrhage and local deposition of cholesterol rich erythrocyte membranes could promote lipid core expansion and atherosclerosis.^29^ In addition, it was proposed that the erythrocytes could facilitate cholesterol uptake^30^ as red cell membrane cholesterol concentration is in equilibrium with plasma levels. Elevations of plasma cholesterol could lead to higher red cell membrane cholesterol content and thus reduced stability and deformability of erythrocytes,^31^ leading to increased turn-over of the red cells. The plasma levels of HDL-C and LDL-C could be a factor that determines the life span of the red cells.^31,32^ Besides, a variety of inflammatory cytokines, such as several interleukins and tumour necrosis factor-α (TNF-α), play key roles in coronary calcification.^25^ Inflammatory factors could affect the iron metabolism of the erythrocytes^33^ and inhibit erythroid cell maturation, thus leading to high RDW. However, the results persisted even after adjustments for CRP, which indicated that RDW may be associated with coronary atherosclerosis through other pathways besides inflammation. It is also possible that high RDW is an unspecific marker of poor general health. RDW might reflect multiple factors contributing to CV disease risk,^34^ which could be associated with calcification processes, but the underlying mechanisms need to be explored.

The red cells survive approximately 120 days in the circulation and during this time, the red cells gradually shrink and become smaller.^35^ High RDW could hypothetically be caused by a high turnover of the red cells, with a high production of large immature red cells. In this case, we could expect to find high RDW and high MCV. Alternatively, high RDW could be caused by a reduced elimination of old small red cells from the circulation.^35^ It has been proposed that reduced production and decreased elimination of the red cells is part of a physiological adaptation to poor health and stress,^35^ which possibly could fill the purpose of saving energy and iron to the body. In the latter case, high RDW would mainly occur in combination with low MCV. The relationship between RDW and CACS was significant in individuals with high MCV above median and the relationship in those with low MCV was weaker and non-significant after adjustments for risk factors. It could therefore be speculated that the association between RDW and CACS might be related to an increased production of new large red cells, rather than reduced elimination of old cells from the circulation. However, more research is needed about RDW in relation to erythrocyte life span to assess this hypothesis.

Previous studies on associations of red cell indices with CACS burden are limited; our results provide new evidence that RDW is associated with CACS burden in the general population and could act as a useful, although unspecific tool for CV disease risk evaluation. One question is whether RDW could add information about coronary calcification in addition to established risk factors. The area under curve statistics improved slightly (from .773 to .776) when RDW was added on top of multiple CV risk factors. Although this improvement seems to be modest, it should be kept in mind that RDW is an unspecific marker that is associated with increased risk of several outcomes besides high CACS.^2-6^

RDW is an inexpensive measure, which is available in most hospital laboratories. Low biological variability is a very important pre-requisite for a biomarker used for screening or risk assessment. Single-measure ICC for RDW was .76 after 1 year in this study, which is comparable to the biological variability for other major biomarkers.

Our findings, together with results from previous clinical studies, suggest that RDW could be a useful, although non-specific marker of the overall CV burden.^34^ However, more work is still needed with respect to standardization of the RDW measures and development of reference values.^3^

### Strengths and Limitations

The large population-based cohort with a high participation rate and information of RDW and coronary atherosclerosis from CT scans are important strengths of the study. We used the RDW-SD^1^ rather than RDW-coefficient of variation, which has been used in many previous studies. RDW-SD reflects an absolute measure of the dispersion of red cell volumes and is independent of the MCV-value. CACS was obtained according to standard methods.^15,16^ It provides accurate and reproducible measures of coronary atherosclerosis, which is a strength of our study. However, many coronary plaques are not calcified and these plaques are not included in the present study. Another strength of the present study is the standardized and detailed protocol with information about the major known atherosclerotic risk factors.

Our study has several limitations. First, this is an observational study and thus unable to establish causality. Elevated RDW could be due to macrocytosis or microcytosis, which in turn could be caused by anaemia due to deficiency of iron, vitamin B12 or folate. We did not have information about blood levels of these nutrients. However, the positive associations remained after exclusion of individuals with supplement intakes of iron, vitamin B12 and folic acid. Haemoglobin disorders and other rare causes of anaemia are very unusual in a general European population.^36^ In this study, the total proportion of anaemia was 2.8% and our findings were similar after exclusion of this group. Although we do not have information on specific causes of anaemia, this indicated that anaemia and haemoglobin abnormalities are unlikely explanations for the association between RDW and CACS.

## Conclusions

This study shows that elevated RDW is associated with CACS in the general population. We propose that RDW could act as an easily assessable and reproducible marker of identifying middle-aged subjects with increased CV burden. To further understand the complex and multiple associations between erythrocyte characteristics and CACS, future studies should evaluate the red cell life span and other detailed characteristics of erythrocytes, in relation to atherogenesis.
